# The politics of face and the trouble with race: Exploring relations at the interface between the individual and the collective in forensic practice

**DOI:** 10.1177/03063127231211817

**Published:** 2023-11-17

**Authors:** Amade M’charek, Irene van Oorschot

**Affiliations:** 1 University of Amsterdam, Amsterdam, Netherlands; 2 Erasmus University Rotterdam, Rotterdam, Netherlands

**Keywords:** race, face, forensics, genetics, craniofacial depiction, DNA phenotyping

## Abstract

This special issue interrogates race through the lens of face. Its central faces are those in forensic settings. Promising immediate legibility and access to the individual suspect, forensic faces nevertheless mobilize a variety of collectives. We offer conceptual and methodological tools to examine the face as both an individual and a collective phenomenon, and demonstrate through detailed cases how the face thus allows us to address the absent presence of race. Given its long and convoluted history in physical anthropology, as a marker of racial typology, the face forces us to reckon with colonial histories of ordering human difference. But the face also allows us to question a regime of visuality that congeals around the face in Western culture. In this introduction to *The Politics of Face and the Trouble with Race* we elaborate these concerns and introduce the contributions to this volume.

## The promise of the face

The face, in Western culture, is a miracle. No two faces are the same, we say. The face makes us unique and holds the key to our individuality. Imagined as allowing for recognition, the face is the location of our deepest ethical commitments to each other. Or indeed to the more abstract Other, as a Levinasian ethics would emphasize (Levinas, 1990). Emanating from the uniqueness of the face is an ethics of recognition, of recognizing the depths of the Other. At the same time, however, the face has been historically crucial to imaginaries of racial difference, particularly as these were organized around physical types. In 19^th^- and early 20^th^-century physical anthropology and ethnology, for instance, such differences were made legible and accessible through the depiction of different facial types (M’charek & Schramm, 2020), which representational practices relied on extensive (and often coerced) colonial knowledge practices (Schramm, 2020). The face, then, has a puzzling reality: It is at once viewed as the locus of individuality, and simultaneously taken to provide access to racial types.

In forensics, faces are typically tasked with a rather prosaic purpose. Technologies ranging from eyewitness-based composite sketching to DNA phenotyping based on biological traces collected at the crime scene allow criminal investigators to produce clues as to the likely identity of an unknown suspect (M’charek, 2008; Ossorio, 2006). In these practices, faces are produced less as ethical zones, and more as functional and affective operators in investigations. While their purpose is not the identification of an individual suspect, their power resides in how they produce a population of interest (Cole & Lynch, 2006), a population that, more often than not, is racialized (M’charek, 2020; Ossorio & Duster, 2005). Forensic faces help to narrow down the pool of possible suspects for criminal investigators, but also function as ‘tentacular’ (M’charek, 2020) for the public. Such faces are tentacular because they generate and feed on the responses of the public (‘He looks like a Cape Verdian.’ ‘I feel I know him.’) and evoke affect and concern, and in doing so help to ‘keep the case alive’ (M’charek, 2020; see also Hopman & Bleumink, 2023). Indeed, advances in forensic genetics such as DNA phenotyping, in which DNA traces on the crime scene are mobilized to produce a ‘snapshot’ of the unknown suspect, are increasingly used by investigating authorities (Hopman, 2022; M’charek, 2020; Samuel & Prainsack, 2019; Toom et al., 2022; Wienroth, 2018).

The promise of individuality is highly appealing, but it is typically sustained because we are confronted with these forensic faces after the fact, that is, once they are produced and distributed. Think of the facial depictions or snapshots based on DNA phenotyping and facial morphology (Hopman & M’charek, 2020; Wienroth, 2020) of unknown suspects distributed by police officers in specific communities. Or in the news, when craniofacial forensic experts have finally reconstructed the face of an as-of-yet unknown victim of a gruesome crime. These faces are the end-product of specific practices of face-making, in which routines, materials, infrastructures, classifications, ordinary humans and their designs help to *produce* the face. What if we start our engagement with the face *there*? What if we bracket the promise of individuality that the face offers and instead turn the face into a problem? What knowledges and forms of expertise do practices of face-making require? What objects, instruments, and infrastructures are mobilized? How is the promise of individuality and recognition sustained? Asking this question is an attempt to address the face not as the ‘beginning of a relation’ à la Levinas (M’charek & Schramm, 2020, p. 322), but rather as engendered in and by multiple forms of material-semiotic relatings (M’charek, 2020; M’charek & Schramm, 2020). The face ‘does not stand alone but is a material-semiotic object, enacted through its very relation[s]’ (M’charek, 2020, p. 376).

The contributions to this special issue, tackling a diversity of practices such as DNA phenotyping, genetic facial morphology, computational landmark analysis, spectrophotometry, Principal Component Analysis, and cranio-facial reconstruction, all offer insights into where and how such faces are made. In this sense, we are interested in turning the face from a solution into a Foucauldian problem (Foucault, 1984; see also Deacon, 2000) that urgently requires empirical attention, political engagement, and care. Such forensic faces require care, we suggest here, because the individualizing promise of the face is only sustained in and through the mobilization of multiple, often racialized, collectives. Making a face, the contributions to this special issue show, is a matter of drawing in markers of collectives through reference to sedimented technologies, data infrastructures, and statistical analyses on for instance tissue depths, skin color, and facial shape, and includes largely unexamined conceptions of the normal and the typical.

Involving conceptions of normality and abnormality, the forensic face, then, also requires us to reckon with practices of making, seeing, and ordering human difference. Attending to the face is also attending to race (M’charek & Schramm, 2020), and to how an ‘abstract machine of faciality’ (Deleuze & Guattari, 1987, p. 168) sustains, engenders, and excretes conceptions of human difference, including conceptions of normality and deviance. The normal, the average, and the typical face are accomplishments inflected and informed by classifications of biological difference. Yet race is a fraught and complex phenomenon in these practices. While data infrastructures and taken-for-granted classifications continue to enact racial difference, forensic geneticists are keen to emphasize that they are taking us beyond crude and politically suspect notions of race. Yet race persists, precisely as an *absent presence*, and thus haunts the face (M’charek & van Oorschot, 2019). Problematizing the face (Foucault & Rabinow, 1984), then, also allows us to zoom in on race in these practices. In a counterintuitive gesture, the face and its promise of individuality allow us to interrogate how race figures as an absent presence in practices that seem indifferent to race. Exploring a range of different technologies situated in different places and times, the articles in this volume show how scientific processes of fragmentation and clustering have become part and parcel of more mundane ways of identification, such as through an identity card, as well as our everyday ways of seeing and clustering faces—our everyday ways of doing faces and races simultaneously. The face, we suggest, is always more than one, oscillating between individuality and collectivity (M’charek & Wade, 2020; see also Hopman & M’charek, 2020; Skinner, 2020).

In the following pages we want to situate this problematization of the face within the social study of genetics and forensics, approaches that have highlighted the production of human difference through genetic research. We highlight how the contributions in this special issue build on – but also extend and rearticulate – the social study of forensics, particularly as we reorient attention away from the molecular depths of the body towards the sur/face. In our more in-depth discussion of the contributions in this special issue below, we highlight what forms of address and care race may require. M’charek (2023) examines how race is not simply an *object* multiple, but also operates as *method* and *theory* in these practices, while Granja and Machado (2023) examine how forensic geneticists navigate the fraught epistemological and political character of DNA phenotyping. Second, we also further complicate approaches to biological race, demonstrating the multiple and not necessarily commensurable practices of making and seeing difference in these practices, including conceptions of the normal, the average, and the standard, at work in the practices studied by Jong (2023). Third, this special issue, particularly the piece by Hopman (2023) also emphasizes the inherent historicity of these practices and examines how colonial histories of collecting and ordering are folded into current practices, so that the past stretches into the present. Fourth, this special issue also explores questions of legibility and visuality as they congeal around forensic faces in practice, demonstrating how racial assumptions are built into statistical techniques designed to approach human modes of seeing difference, in the contribution by Nieves Delgado (2023), and experimenting with film montages to extend care to our own (often implicit) techniques of seeing difference, in Plájás (2023).

## From body to face, from molecular depth to the sur/face

In recent decades, scholars in Science and Technology Studies (STS) have developed a strong body of scholarship on the making and ordering of human difference in scientific practices. While emergent genetic and genomic research in the 1990’s and since has promised to move us beyond race, STS scholars have demonstrated the obduracy of race and racism in science and society (Koenig et al., 2008; M’charek, 2005; Reardon, 2009; Whitmarsh & Jones, 2010). STS scholars have emphasized that biological race was not cleanly left behind in the dustbin of history. In fact, the 19^th^-century classifications that current day science seeks to surpass continue to be built into sampling strategies, statistical models and the infrastructures of geneticists. Race, it turns out, is not so much left behind as it is ‘reinvented’ (Abu El-Haj, 2007). Motivated by a concern with the broader performative and political effects of such genetic knowledges, STS scholars examined the potential of genetic technologies to (re)biologize human difference (Duster, 2003; Goodman et al., 2003, Koenig et al., 2008). While we may not be dealing with simple iterations and replications of 19^th^ century racial science and racial typologies, race nevertheless continues to haunt these genetic practices (M’charek & van Oorschot, 2019) as an absent presence (M’charek et al., 2013). To be sure, this absent presence is not simply a matter of (often specifically European) denial of racism, but more precisely also enacted at the level of technologies used, sampling and research procedures (Duster, 2006b; Fujimura & Rajagopalan, 2011; M’charek, 2014), where biological differences are mobilized to produce different collectives. In these practices, these biological and molecular differences are often delegated a status as proxies for race, only necessary in a ‘meantime’ (Kahn, 2012a) until full individualization is realized. As a result of these processes, researchers have convincingly demonstrated that race has shifted shape and had increasingly become molecularized (Abu El-Haj, 2007; Duster, 2006a; Fullwiley, 2007). This molecularization, in which human difference is produced as residing in one’s DNA, represented a novel mode of making and apprehending human difference. Crucially, it also represented a mode of enacting human differences that is at odds with the discarded, 19^th^-century histories of racial typifications that depending on the external physical differences, or the *phenotype*.

How forensic DNA has reshaped the field of policing and identification cannot be overestimated (Hindmarsh & Prainsack, 2010; Lynch & Jasanoff,1998; Lynch et al., 2010; Toom et al., 2022; Williams & Johnson, 2013). Forensic practices have historically been about identification. Initially forensic DNA was about a comparison between the DNA profile retrieved from biological traces found at a crime scene and that of a suspect. However, to establish the uniqueness of a possible match the profile needs to be compared to a reference population, a population that is representative of the specific DNA profile. These populations were often ordered in reference to racialized categories, so that, in its early days, racially defined collectives played a crucial mediating role in the matching of one individual sample to another individual’s DNA (Lewontin, 1992; Lewontin & Hartl, 1991; M’charek 2000). This reference population caused controversies in the early 1990’s that revolved around biases and racial classifications (Chakraborty & Kidd 1991; Kahn 2012b; Lander, 1992; Lewontin & Hartl, 1991; M’charek, 2000). In these identification processes, race assumed an absent present character, hiding out in the statistical models and reference populations in use by forensic scientists. This particular controversy was eventually resolved as a result of the growth of DNA databanks and expansions in the number of genetic markers validated for forensic use, so that DNA profiles became specific for individuals and did not rely on the use of crude population categories anymore (e.g. M’charek, 2008; Toom, 2012).

Meanwhile, developments in forensic genetics continued apace. While forensic DNA had developed into the golden standard of forensics, in terms of its use it had started to shift from an identification technology into an investigative tool. Its aim was not merely to include or exclude a suspect at hand, but also to probe into the identity of an unknown suspect, to generate a suspect. To this end, DNA is used as a clustering technology to reduce the pool of interesting people for investigating authorities. Precisely this use has moved race from a category hidden in the technologies of forensic DNA to a productive tool to delineate populations of interest (Kahn, 2012a; M’charek, 2008; Sankar, 2012). Concretely, technologies such as DNA phenotyping, forensic facial morphology, and the inference of biogeographic ancestry all rely on racialization as the most relevant clustering tool for criminal investigators. These technologies have produced a renewed interest in the surface of the body, an interest that M’charek (2020) calls a return of the phenotype. This involves a biologization of appearance and, given the context in which these technologies are used, a biologization of race at both the molecular *and* the surface level. For instance, familial DNA searching and ancestry searching in forensic genetics are emerging as crucial investigatory instruments for police actors (Jong & M’charek, 2018; M’charek, 2020; Van Oorschot & M’charek, 2021, 2022). In such cases, genetically produced collectives are typically brought into relation with everyday categorizations of externally visible characteristics (e.g. ‘European’, ‘white’, ‘Turkish’), bringing about the possibility for further racialization of crime and marginalized communities (Van Oorschot & M’charek, 2021, 2022). Genetic forensics, then, powerfully informs not only the police search for an individual suspect, but also has the capacity to co-shape broader imaginaries of belonging, descent, and deviance. These studies demonstrate that in 21^st^ century forensics, race is not simply about biology, but rather is a nature-culture assemblage, involving both biology and social and cultural markers. It is relevant in this context that genetic practices, within and beyond forensics, have been active in generating and coproducing novel imaginaries of belonging, autochthony, descent, and national selves (Fortier, 2011; M’charek et al., 2014; de Rooij et al., 2014; Santos & Maio, 2004; TallBear, 2014; Watt & Kowal, 2019). The complex clusterings and classifications of groups of people, through scientific or social technologies, combined with the legacies of slavery, colonialism, and migration, all have the capacity to feed into the production of race in forensic settings. Indeed, forensics is a prime case to think through the production of race as a matter of both biology and culture, as it is a field that relies on an intensive circulation between science and society when it comes to material objects and knowledges.

Novel technologies in forensics and fast-paced forensic developments require us to attend not only to the (supposed) molecular depths of the body and the racialization of such genetic differences. We must also reinvigorate a critical and careful concern with the production of the *surface* of the body, and inquire into the ways externally visible characteristics are informed by, and in turn shape, novel conceptions of sameness and difference and normality and abnormality. Secondly, these developments also draw attention towards the face. Productive in the making of individual suspects as well as in the drawing out of suspect populations, the face is positioned at the cutting edge of forensic innovation. As various contributions in this special volume show, however, the ways in which faces are made and facial recognition—seeing—takes place are shot through with histories textured by biological classifications. Here, too, the contributions to this special issue show, race rears its head.

## Giving the face power to make us think

So what is there to learn from and with practices of face-making about this slippery, absent present object of race? The contributions to this special issue take on this urgent question. They provide ample opportunity to rethink taken-for-granted approaches to face, as well as advance contemporary approaches to the question of race in forensics. Indeed, problematization is here a gesture that precisely consists in giving face, particularly face-making practices, the power to make us think rather than recognize (Stengers, 2005), to experiment with our methodological and conceptual tools, and to test our sensibilities.

### The difficult paths of generosity

In her piece in this edited volume, ‘Curious about race: Generous methods and modes of knowing in practice’, M’charek (2023) explores what we can learn about race by attending to the face and practices of face-making, and crucially, what mode of attention we could bring to the fraught realities of race. While earlier approaches to the production and making of race have demonstrated its multiple, relational, and absent-present character, her analysis takes this mode of analysis a crucial step further by not focusing solely on the multiplicity of race as an object, but on the different ways it operates. Sometimes race is an *object* (of research, of concern, of attention). Yet at other times it rather operates as a *method*: a device for classifying data, a technique of lumping and splitting, an infrastructure of sameness and difference. And yet at other times race operates as a *theory*, rendering difference intelligible and perhaps: manageable. Together, these three dimensions make up what she coins an *affinity concept*, here conceptualized as a concept that ties these different dimensions and operations together. Historically tracing and disentangling these three operations of the affinity concept ‘race’, and extending this analysis into the present, her contribution is also a call for a *generous method* of engaging with race and its multiple operations across sites and contexts, including practices of forensic geneticists. Generous methods, she argues, ‘embody an ethos of “getting out of our way” for our objects of study giving [them] our time and attention’, and inviting ‘a material semiotic take on our objects of study, to view them not as singular entities, but as inherently relational objects that have the capacity to shift and change depending on the practices in which they happen to figure’ (M’charek, 2023). Extended to the troublesome, difficult, perhaps even ‘ugly’ object/method/theory of race, such methods require the difficult and circuitous work of tracing relations and presences, as well as attuning oneself to things made absent and histories glossed over.

Where M’charek equips us with methodological and conceptual sensibilities to track the multiple operations of race within (forensic) genetics, the contribution by Granja and Machado (2023), ‘Forensic DNA phenotyping and its politics of legitimation and contestation: Views of forensic geneticists in Europe’ explores how forensic geneticists in Europe navigate the troubled political and epistemological terrain of the application of forensic phenotyping technologies. As DNA phenotyping is a novel and emerging technology, it is mired in questions about its social legitimacy, legal efficacy, and political consequences and part and parcel of ongoing traffic between legal and scientific communities and practices. Offering a sophisticated account of the multiple forms of (discursive) boundary work in which these forensic geneticists engage to ensure the legitimacy of DNA phenotyping in the European context, the article develops not only a conceptual understanding of these negotiations and contestations by situating these in broader biolegality approaches (Lynch & McNally, 2003), but can also be read as an important tool for other researchers interested in navigating the field of forensic genetics. Characterized by practices of boundary work that highlight contrasts between the criminal justice system and science, between experts and non-experts, and between ‘good’ and ‘bad’ science, these forms of boundary work can also be read as clues to the affects and sensibilities that shape this field of practice.

### Materialities and temporalities and the making of the face

The two contributions, one by Hopman, the other by Jong, each take up the challenge to study race by attending to practices of face-making. Drawing on the notion of the ‘folded object’ (M’charek, 2014), Hopman’s (2023) contribution in this volume, ‘The face as folded object: Race and the problems with ‘progress’ in forensic DNA phenotyping’, reveals how 19^th^- and 20^th-^ century histories of racial typology and classification shape and inform contemporary practices and technologies. Drawing on ethnographic fieldwork across different genetics laboratories, she demonstrates how the data practices that feed into the development of DNA phenotyping, including the ordering of skin color data taken with a spectrophotometer, the analysis of facial shapes using computational landmarks, and the collection of iris photographs, reproduce racial types as well as racially inflected averaging statistics as developed by Quetelet and Galton. The individual face, in her analysis, becomes a fold of these multiple histories—but it also promises a particular future of ever greater specificity and individuality. Emphasizing the continuing presence of racial orderings in these practices, her contribution challenges promissory and progressive temporalities, and asks us to attend to folded—and often half-forgotten—histories. Indeed, engaging with the face also means engaging with histories of racial classifications and their agency in the here-and-now. In this sense, engaging with the face also means rethinking progressive temporalities as they are evoked in the practices we study, and mobilizing topological approaches to time that can trace and track how histories are folded into present practices and objects.

In her contribution ‘On the persistence of race: Unique skulls and average tissue depths in the practice of forensic craniofacial depiction’, a prizewinning article, Jong (2023) takes on another practice of face-making: that of craniofacial construction, in which unknown victims are ‘given an individual face’ in order to facilitate criminal investigation. These practices, involving a great deal of craftmanship, mobilize contested ancestry classifications, which are used to gauge a set of likely markers of difference, such as tissue depth at different locations on the face. As a field with a particularly fraught history with race, this field also tries to ‘move away’ from race, yet it remains present in the population data it needs to employ to make a face. Tracing how these forensics experts manage this field of tension between individual faces and collectives, Jong develops an approach to race as a ‘translation device’ that, in these practices, is approached with a high level of concern and care. While population data may provide helpful direction in the crafting of an individual face, it is never quite enough to approach the individuality the face has to convey. The materiality of the skull itself often requires experts to calibrate and adapt these population data. Here, population data are not destiny. If we read Jong’s and Hopman’s articles as in conversation, they suggest that histories of racial classification play a role, yet that these classifications do not solve but rather defer the question of individuality. Together, Hopman’s and Jong’s contributions can be read as accounts of the ways difficult and fraught histories are folded into objects and infrastructures, which, in practice, are both mobilized and contested. We note quite proudly that Jong’s article has received the Nicholas C. Mullins prize from the Social Study of Science Society.

### Seeing face, seeing race

Learning about race by attending to the face, this special issue also investigates practices of *seeing* face and seeing race. If difference is relational, potentially multiple, and in the making, we must extend curiosity and care to the operation of seeing difference. Bringing to light the largely implicit conceptions of seeing and recognition of the normal and the abnormal at work in these technologies and forensic practices, two contributions in particular raise the question not only about what racial ontologies of normality are produced, but also how seeing itself partakes in the production of racialized faces. Such modes of seeing are informed by an historically produced and cultivated ‘sensorium’ (Gilroy, 1998) in which some differences register with some immediacy as different, which operation also informs forensic and even legal modes of seeing and recognizing difference (van Oorschot, 2020). But how is this sensorium mediated? What objects, infrastructures, technologies and orderings contribute to the racialized seeing of faces? And what ontologies come into play? The contribution by Nieves Delgado (2023), ‘Race and statistics in facial recognition: Producing types, physical attributes, and genealogies’, focuses attention on the implication of a highly specific statistical technique, Principal Component Analysis (PCA), that is at the core of both facial identification technologies and composite sketching systems. Recognizing individual faces (in facial identification technologies) or producing individual faces (in composite sketching) depends on the accurate mapping of facial similarities and differences. Yet such practices often proceed on the basis of a ‘normal face’, to which identifying idiosyncrasies then may be added. But how is this ‘normal face’ produced? And what is the link between normality and race? Tracking PCA across three cases, Nieves Delgado shows how the ‘normal faces’ produced in and through PCA align themselves with racial expectations. Yet race is not singular in these practices: It features variously as a racial type, as a physical attribute, or as ancestry. Once the individuated face is opened up, we encounter race again – in all its multiplicity. Plájás (2023), in her highly experimental piece ‘InterFaces: On the relationality of vision, face and race in practices of identification. A multimodal intervention’, invites you to extend curiosity to your own modes of seeing difference, drawing together film and the written word to craft a multimodal intervention. Drawing on the technique of the collage, the short film that accompanies her text takes the reader through three settings in which face and race become entangled. Reflecting on her own cinematographic work, Plájás seeks to render visible, as well as interrupt, the taken-for-granted visual immediacy we associate with ‘seeing difference’. With deliberate use of the affordances as well as limitations of the camera’s eye, her intervention highlights the translations and solidifications crucial to the production of a forensic composite sketch, meditates on the racial epidermal schema at play in the recognition of others as strangers, and queries the affective and material mediations at work in the production of racialized Gypsy identities. Bringing together these fragments, her text and short film examine, question and aim to undo the taken-for-granted legibility of the face as well as the stability and solidity of biological race. Experimenting in this way, her article also takes us beyond a text-centered focus and explores the crucial role of other media in the making (and unmaking) of the social realities we inhabit.

Taking together, the contributions in this special issue offer a wide variety of cases and concepts. They offer an invitation for others to attend to the face and to interrogate it as crucial surface through which race gets made.
